# Role of Gender in Regulation of Redox Homeostasis in Pulmonary Arterial Hypertension

**DOI:** 10.3390/antiox8050135

**Published:** 2019-05-16

**Authors:** Ruslan Rafikov, Joel James, Nolan McClain, Stevan P. Tofovic, Olga Rafikova

**Affiliations:** 1 Division of Endocrinology, University of Arizona, Tucson, AZ 85721, USA; ruslanrafikov@email.arizona.edu (R.R.); joeljames@deptofmed.arizona.edu (J.J.); nolanm@email.arizona.edu (N.M.); 2 Department of Pharmacology and Chemical Biology, Vascular Medicine Institute, University of Pittsburgh, Pennsylvania, PA 15260, USA; tofovic@pitt.edu

**Keywords:** pulmonary hypertension, gender difference, redox homeostasis, oxidation, antioxidants

## Abstract

Pulmonary arterial hypertension (PAH) is one of the diseases with a well-established gender dimorphism. The prevalence of PAH is increased in females with a ratio of 4:1, while poor survival prognosis is associated with the male gender. Nevertheless, the specific contribution of gender in disease development and progression is unclear due to the complex nature of the PAH. Oxidative and nitrosative stresses are important contributors in PAH pathogenesis; however, the role of gender in redox homeostasis has been understudied. This review is aimed to overview the possible sex-specific mechanisms responsible for the regulation of the balance between oxidants and antioxidants in relation to PAH pathobiology.

## 1. Introduction

Pulmonary arterial hypertension (PAH) is a complex disease involving the proliferation of pulmonary arterial vascular cells that leads to increased pulmonary pressure and eventual right-sided heart failure. Numerous epidemiological studies report PAH to be highly prevalent in women [1], with an approximate female to male ratio of 4:1 [2,3]. In contrast, male PAH patients show a more pronounced impairment of the right ventricular function and experience poorer outcomes [4]. The particular mechanisms responsible for this sex disparity are still not fully understood, and despite numerous research done, no clear consensus has been gained. Nevertheless, not only prevalence and prognosis but also the response of males and females to different types of PAH therapy was found to be gender-specific, highlighting the absolute need to understand the reasons underlying this gender disparity, as well as the need to elaborate the effective gender-specific approaches for PAH treatment.

Oxidative stress is an important and well-recognized pathogenic stimulus that alters multiple pathways and contributes to PAH initiation and progression on several levels. The contribution of gender in redox homeostasis has recently been recognized and described for many different pathological conditions [5,6]. In general, males show higher levels of oxidative stress and less activity of antioxidant enzymes [7,8,9], which may explain the more progressive form of PAH and poorer survival prognosis in males. The protection against oxidative stress seen in females has been historically attributed to the antioxidant properties of female sex hormones; however, recent discoveries propose a contribution from genomic factors as well. Indeed, some gender disparity in the expression or activity of antioxidant proteins was reported to be present even in castrated animals [5]. In humans, the hormone replacement therapy does not decrease the risk of cardiovascular disease in post-menopausal women [10,11]. Finally, the distinct male/female differences in redox homeostasis have been described in neonates, and even in mothers of newborn girls and boys [8].

**Figure 1:**
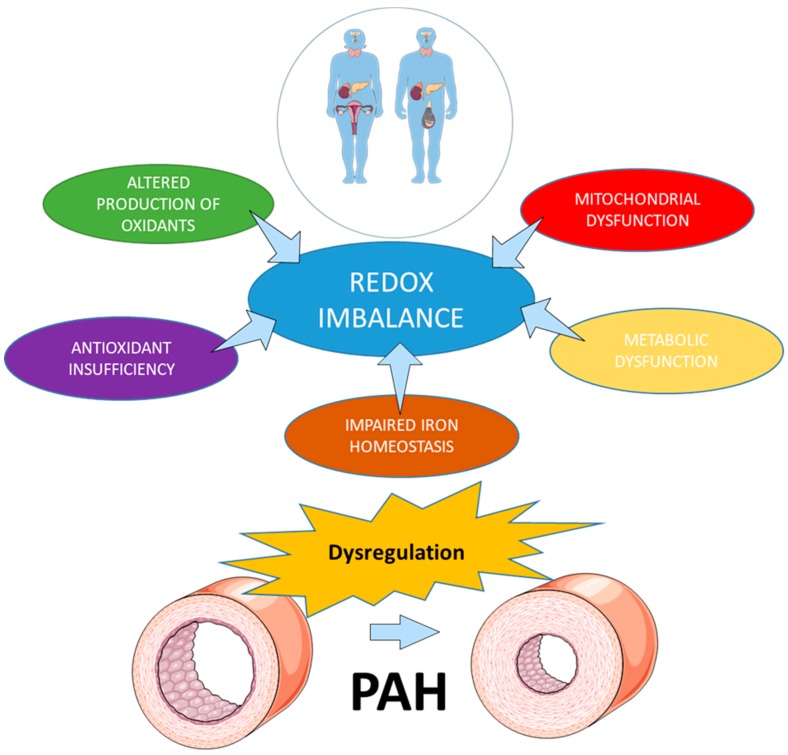
Mechanisms involved in the impaired redox homeostasis in pulmonary arterial hypertension (PAH). Multiple mechanisms involved in redox regulation are affected by gender and could contribute to the development and progression of PAH.

In this review, we aimed to unravel the role of gender in redox regulation and discuss its potential contribution to PAH pathobiology (Figure 1). In particular, we will focus on summarizing the current knowledge regarding the hormonal regulation of redox homeostasis in both genders, role of gender in disease-mediated disbalance between oxidants’ production and antioxidant protection, and gender difference in pathogenic mechanisms responsible for the impaired redox homeostasis, such as mitochondrial and metabolic dysfunction, and iron homeostasis. Understanding the mechanisms underlying the sex differences could result in more precise therapeutic approaches and significantly advance the disease outcome.

## 2. Redox Homeostasis in Health and Disease

Although reactive oxygen species (ROS) were thought to be byproducts of mitochondrial electron transfer reactions or enzymatic reactions, it was discovered that ROS have many biological functions including cell division, apoptosis, and cell defense [12,13]. ROS, such as superoxide (O_2_**^•−^**), hydrogen peroxide (H_2_O_2_), and hydroxyl radical (**^•^**OH), and reactive nitrogen species (RNS), such as nitric oxide (NO**^•^**) and peroxynitrite (OONO^−^), have central roles in immunity and serve as a repertoire of ammunition for the destruction of invading pathogens. More importantly, these molecules serve as messengers, which control and modulate cell signaling pathways and, thereby, govern cellular fates. At physiological concentrations, ROS and RNS have been shown to have indispensable functions in cellular biochemistry and physiology [14]. However, when the amount of ROS/RNS exceeds physiological levels, these radicals start initiating oxidative/nitrative stresses that are deleterious for cells. Therefore, the precisely controlled and balanced production of free radicals is essential for normal cell function. Several levels of antioxidant defense consist of (1) antioxidants that coherently neutralize O_2_**^•−^_,_** the primary ROS produced in the cell, and H_2_O_2_ formed in the dismutase reaction or by cell oxidases, and (2) the molecules with free radical scavenging activity. Thus, the enzymatic antioxidant protection in lungs consists of superoxide dismutases (SODs), catalase (Cat), glutathione peroxidase (GPx), and additional antioxidants such as heme oxygenase-1 (HO1), redox proteins including thioredoxins (TRXs), peroxiredoxins (PRXs), and glutaredoxin. Besides, the redox homeostasis is also maintained by ROS scavenging antioxidant compounds that are either hydrophilic and, therefore, control the levels of ROS in cytosol (ascorbic acid, uric acid, and glutathione) or lipophilic, and work to prevent the oxidation of lipids in membrane compartments (alpha-tocopherol (vitamin E) and ubiquinol).

**Figure 2:**
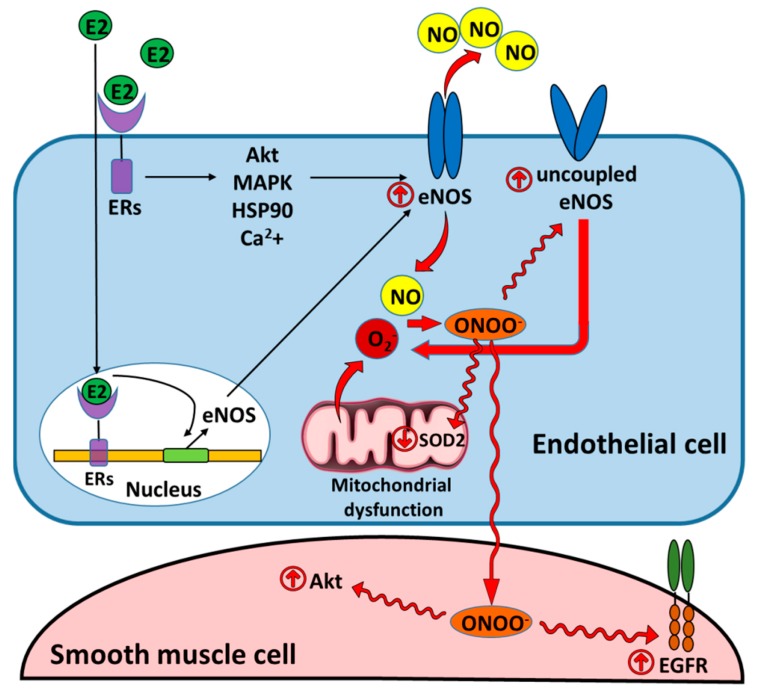
Estradiol increases NO bioavailability that could contribute to the development of nirosative stress and promote pulmonary hypertension. The genomic and non-genomic mechanisms of estradiol-mediated synthesis of NO increase NO bioavailability and ensure cardioprotective effects of NO. However, oxidative stress induced by mitochondrial dysfunction or uncoupling of endothelial nitric oxide synthase (eNOS) results in the increased formation of peroxynitrite responsible for the persistent nitrative stress and posttranslational modifications of essential cellular enzymes in endothelial and smooth muscle cells. These modifications include inhibitory nitration of eNOS and superoxide dismutase 2 (SOD2) that promotes the redox misbalance [21,22,23], or activatory modifications of Akt and epidermal growth factor receptor (EGFR) that stimulates uncontrolled proliferation of SMC [24,25]. E2: estradiol; ERs: estrogen receptors; ONOO^−^: peroxynitrite; MAPK: mitogen activated protein kinase; HSP90: heat shock protein 90.

Nevertheless, stringent control is sometimes lost when the rate of ROS/RNS production transcends the limits of these protective mechanisms. The disruption of redox homeostasis leads to untoward effects such as glutathione oxidation, protein carbonylation and thiolation, lipid peroxidation, protein tyrosine oxidation and nitration, and DNA damage. These processes are known to disrupt cellular signaling and homeostasis, trigger cellular damage, and activate an inflammatory response. The inflammatory cells that migrate to the site of damage massively release the ROS during the “respiratory burst,” which additionally potentiate oxidative stress and tissue injury. This feedforward mechanism is currently considered to be responsible for the chronically impaired redox homeostasis and contributes to the pathogenesis of many cardiovascular diseases, including PAH. In our recently published research [15], we confirmed that the type of cell death (necrosis versus apoptosis) significantly affects redox homeostasis and PAH outcome. Since apoptotic cells are known to release the factors in the already pre-oxidized state [16], they do not activate inflammation and die by an “immune-silent” type of cell death without potentiation of tissue damage. In contrast, necrotic cells spill their content in the original, reduced, state into the extracellular environment. This, in turn, alters redox balance outside the cell and prolongs the activity of redox-sensitive damage-associated molecular patterns (DAMPs) that work as potent activators of inflammatory response and mediators of severe inflammation [17]. There is a gender difference in the type of cell death in response to the damaging stimuli, with more necrosis associated with the male gender [18]. The subsequent activation of oxidative stress and inflammatory response in males coupled with the less effective antioxidant protection may provide a solid background for the poor survival prognosis seen in males with PAH and other inflammatory diseases [19]. Moreover, this disparity in redox homeostasis could be responsible for the gender-specific manifestation of PAH [20] (Figure 2), as it will be discussed later.

## 3. The Role of Sex Hormones in Redox Homeostasis and Pulmonary Arterial Hypertension

Female sex hormones, estrogens, are potent antioxidants that control the level of ROS in different cells types. The classical mechanism of action involves binding to estrogen receptors α and β (ERα and ERβ) in the nucleus, which induced receptor’s dimerization, and binding to estrogen-response elements located in the promoters of target genes [26]. This classical pathway is responsible for the regulation of expression of multiple antioxidant proteins and controls antioxidant defense in cells. Estrogen receptors can also regulate the gene expression without direct binding to DNA through a protein–protein interaction with transcription factors. Finally, activation of plasma membrane-associated ERα, ERβ, and G-protein coupled ER (GPER, GPER1) mediates non-genomic actions of estrogens, which could also stimulate different signaling pathways, for example, activation of pro-survival mechanisms or production of “good radical,” nitric oxide (NO) (Figure 2).

The protective effect of estrogens on cardiovascular function mediates through several mechanisms that include reduced expression or activity of ROS producing enzymes increasing expression and increased activity of antioxidant enzymes in different cell compartments. Elevated production of NO mediates vasodilatory, anti-thrombotic, and anti-inflammatory properties. Beneficial effects of estradiol and some of its metabolites are due to the activation of pro-survival pathways or their anti-inflammatory, anti-apoptotic, antioxidant, and anti-fibrotic properties. These effects could play a pivotal role in protecting female hearts from failing. Indeed, a number of clinical studies have confirmed that despite the high prevalence of females in PAH, the female gender is generally associated with much better survival compared to males. Our group has discovered that poor survival rate in male rats treated by combination of Sugen 5416 and hypoxia (Su/Hx) correlated with a severe right ventricle (RV) dysfunction and RV fibrosis, while in female rats, despite the similar elevation of RV systolic pressure (RVSP), the hearts stay preserved during the whole 14-week period of study [20]. Other studies in the same Su/Hx, angio-proliferative model of PAH [27], hypoxia-induced PAH [28], and monocrotaline (MCT) model [29,30,31] show that estrogen therapy improves RV function, while hormone depletion worsens the disease. The therapeutic effect of estrogen was found to be due to the stimulation of cardiopulmonary neoangiogenesis, and suppression of inflammation, fibrosis, and RV hypertrophy [29].

Nevertheless, the prevalence of females in PAH is known to be significantly higher compared to males. This phenomenon, known as the “estrogen paradox” [32], remains unsolved, even despite significant progress in uncovering the complex contribution of estradiol and its metabolites in the pathogenesis and progression of PAH [32,33,34]. Thus, the association between increased estrogens taken as oral contraceptives, as hormone replacement therapy or during pregnancy, and PAH has been previously suggested. Although further research is required for uncovering this mystery, we recently proposed the “three-tier-effects of estrogens” concept that takes into consideration effects of estrogens in pulmonary vasculature (intact versus highly proliferative) and in the right ventricle in the absence or presence of oxidative stress and inflammation [35]. Besides, we propose that the protective and pro-survival properties of estrogens remain a double-edged sword in the situation when the disease progression is associated with cell over-proliferation [32]. Thus, the increased production of female sex hormones is associated with many cancers [36]. PAH that is currently viewed as a disease with a “cancer-like” uncontrolled proliferation of pulmonary vascular cells [37] could become additionally promoted by pro-mitogenic properties of estrogens. Indeed, estradiol and some of its metabolites, like 16α-hydroxyestradiol, possess strong anti-apoptotic, proliferative, and angiogenic properties [33]. Therefore, inhibition of estrogens production could be beneficial in PAH patients [38].

By activating the rapid, non-genomic, membrane-associated responses, estrogens trigger vascular cell proliferation through mitogen activated protein kinase (MAPK) and phosphatidylinositol 3-kinase (PI3K)/Akt pathways [39]. The last signaling cascade is also responsible for estradiol (E2)-mediated stimulation of endothelial nitric oxide synthase (eNOS) in vascular cells [40] (Figure 2). Although under normal conditions NO plays an important vasoprotective role, under oxidative stress conditions NO could react with O_2_^−^ to produce a potent oxidant and nitrative agent, peroxynitrite (ONOO^−^). ONOO^−^, in turn, mediates posttranslational modification of many proteins [21] including those responsible for growth activation. Thus, our group has previously reported that ONOO^−^ induced oxidation of epidermal growth factor receptor (EGFR) [25] or posttranslational nitration of Akt [24] results in uncontrolled over-activation of both proteins and increased proliferation of vascular cells (Figure 2). Although it seems that this mechanism can be an important regulator of vascular cell repair in case of the occasional burst of ROS production, under conditions of chronic oxidative stress seen in PAH, the entire balance could become shifted toward an ongoing proliferation and promote remodeling of pulmonary arteries.

**Figure 3:**
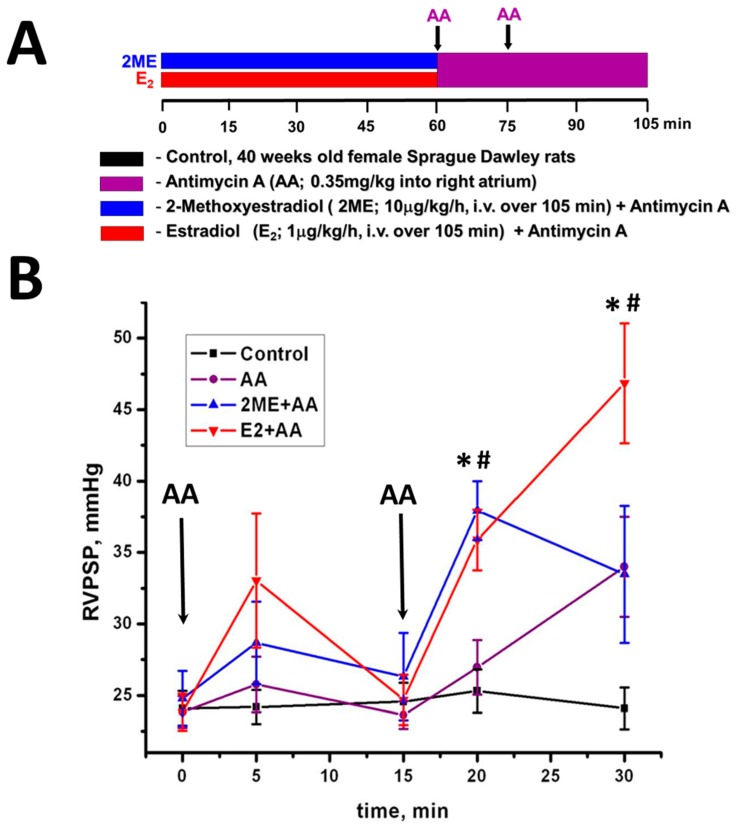

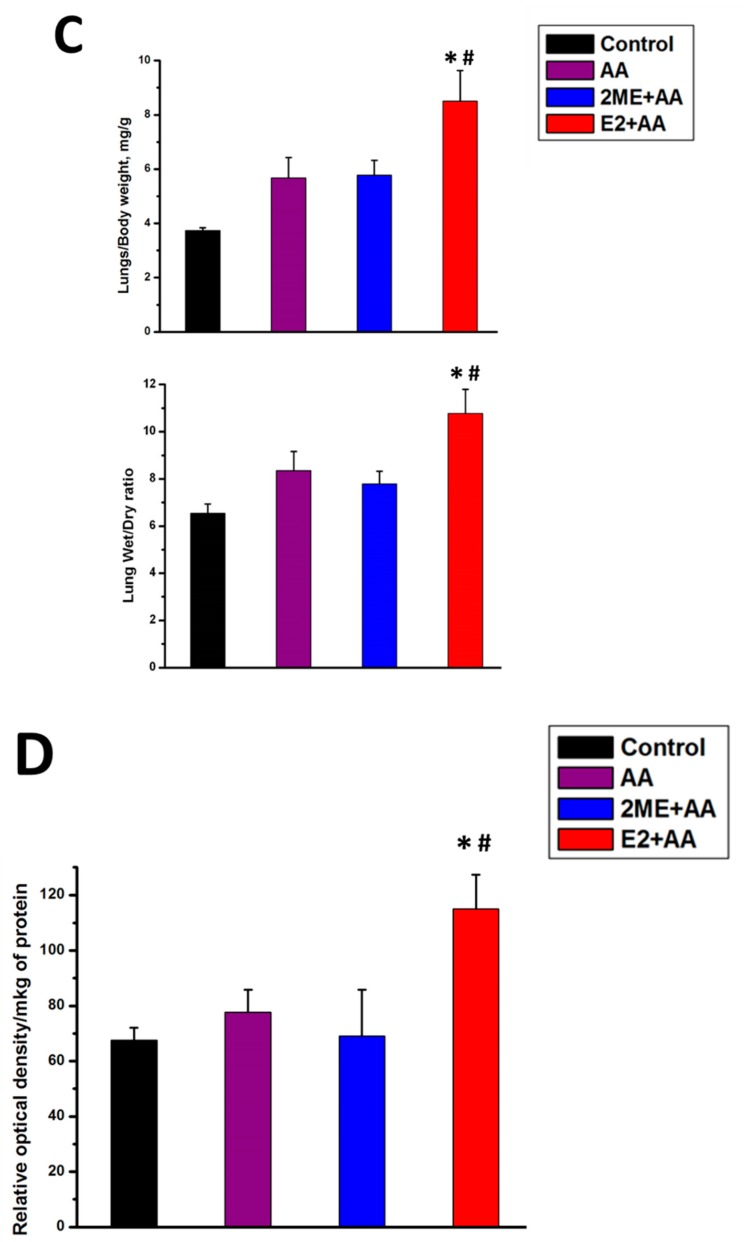
Estradiol supplementation on the background of oxidative stress induces pulmonary vasoconstriction and vascular damage. (**A**) An experimental protocol. Sprague Dawley female rats 40-week old were obtained from Charles River Laboratories (Wilmington, MA, USA). Animals were kept in a standard 12-h light/dark cycle and received standard rodent food and water ad lib. All experimental procedures were approved by IACUC of University of Pittsburgh (ethical protocol code: #0808076 (2008) and #13111158 (2013)). On the day of surgery, animals were anesthetized (Inactin, 100 mg/kg i.p.) and the pressure transducer catheter (SPR-513, Millar Instruments, Houston, TX, USA), connected to a Millar Transducer Control Unit TC-510 and PL3504 PowerLab 8/35 data acquisition system (ADInstruments, Colorado Springs, CO, USA), was inserted into right ventricle (RV) via the right jugular vein and right atrium as previously described [43]. Two PE-50 polyethylene tube catheters filled with 0.9% saline were inserted into the right jugular vein and further advanced to the right atrium for either 2-metoxyestadiol (2ME)/E2 or Antimycin A (AA) injections. The animals were randomized in 4 experimental groups: 2ME and E2 groups received a continuous delivery of 2-metoxyestadiol (2ME, 10 µg/kg/h) or estradiol (E2, 1 µg/kg/h) correspondingly during the entire period of study (105 min). Sixty and 75 min after experiment initiation, the bolus injection of selective inhibitor of mitochondrial complex III, Antymicin A (AA, 0.35 mg/kg as previously published [43]) was given to 2ME, E2, and AA animal groups. AA group received vehicle instead of 2ME or E2. Control group received vehicle only during the entire experiment. At the end of 105 min experiment, the animals were euthanized and the lungs were collected for the analysis. (**B**) Right ventricle peak systolic pressure (RVPSP) in all animal groups recorded after initiation of AA treatment. (**C**) Pulmonary edema assessed by measuring wet lung weight normalized per body weight and wet/dry lung ratio. (**D**) The total level of protein nitration of the main pulmonary artery. *N* = 4–8; * *p* < 0.05 vs. Controls; # *p* < 0.05 versus AA group.

Besides, a severe acute oxidative stress on a background of enhanced NO bioavailability could also be deleterious. For example, inhibition of mitochondrial electron transport chain (ETC) is known to produce severe oxidative stress due to a leakage of the electron on oxygen [41,42]. By using a specific inhibitor of the third complex of ETC, Antimycin A (AA), we have induced a severe acute oxidative stress in lungs of one-year-old female rats that were pre-treated, or not, with E2 and its non-estrogenic metabolite, 2-metoxyestadiol (2ME) (Figure 3A). Interestingly, AA+E2 group showed an increase in RV peak systolic pressure (RVPSP) that becomes significant compared to the control and AA only group after the second injection of AA (Figure 3B). Importantly, only animals pre-treated with E2 had significant lung edema (Figure 3C) and increased level of protein nitration in the pulmonary artery (Figure 3D). We conclude that elevation in ROS production due to the treatment with AA alone or with AA and 2ME, which is incapable of stimulating eNOS, induces no nitrosative stress. However, co-treatment of AA with E2, which increases NO bioavailability, elevates ROS together with NO and initiates severe nitrosative stress. This nitrosative stress is more damaging compared to oxidative stress, since it damages pulmonary endothelial cells, disrupts the endothelial barrier, and induces pulmonary edema. Moreover, the severe damage of pulmonary endothelium could be responsible for an acute vasoconstrictive response.

A similar effect has been described by our group in young female rats with intact ovary function [43]. The chronic inhibition of mitochondrial respiratory chain induced a progressive accumulation of nitration proteins in the lungs of young female rats, which correlated with pulmonary edema, a significant increase in RVSP, and pulmonary vascular remodeling. Importantly, not only in rats but in female PAH patients, the amount of circulating nitrated proteins has significantly correlated with PAH progression. No correlation has been found in males. Thus, increased nitrative stress can be a significant determinant of PAH initiation and progression, and increased NO bioavailability in females can put female gender at a greater risk.

**Figure 4:**
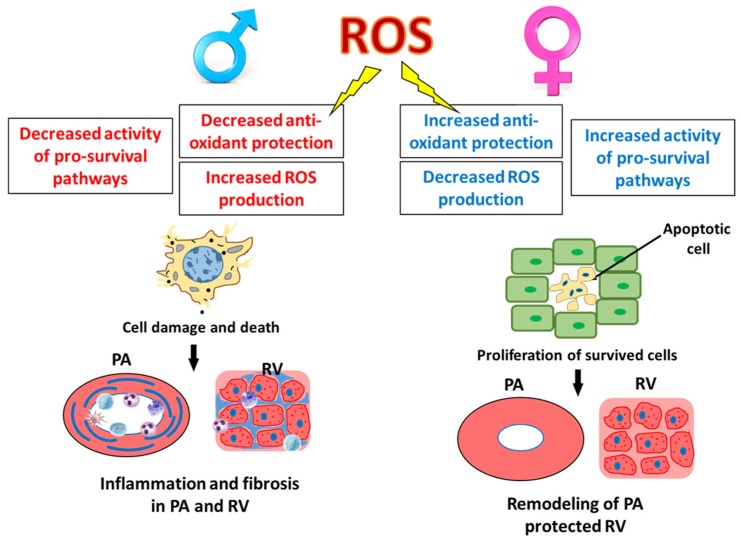
Gender-specific outcome of oxidative stress. The lower antioxidant defense system and decreased pro-survival mechanisms promote severe, caspase-independent type of cell death [15,55], and activation of inflammatory signaling and fibrotic changes in pulmonary arteries and RV of males [15,20] (**left panel**). In contrast in females, the intense antioxidant protection and increased pro-survival pathways induce apoptotic cell death [55], increase proliferation of the survived vascular cells, and promote pulmonary vascular remodeling, while preserving RV function (**right panel**).

In contrast, the male gender is associated with higher production of oxidants and lower activity of the antioxidant system. Although reports about the role of androgens in the cardiovascular system are even more contradictory than for estrogens, many of them point toward the important contribution of testosterone and its metabolites in ROS generation [44,45,46,47]. Moreover, testosterone treatments were discovered to abolish the protective effects of estrogen against oxidative stress in animals [48] and women [49]. This increased amount of ROS produced on the background of low levels for all primary antioxidant enzymes such as superoxide dismutase (SOD), catalase (CAT), and glutathione reductase (GPx) induced severe oxidative stress-mediated damage in male tissues [50] and is responsible for the higher level of oxidative DNA damage seen in males [51]. Indeed, previous studies showed that an increase in hydrogen peroxide generation [52] or mitochondria-dependent ROS production [53] stimulated by testosterone promotes cell death. The untreated males or males that were castrated and subjected to acute testosterone infusion were discovered to have a significantly attenuated activity of protein kinase B (Akt) and downstream pathways as compared to male castrate or males with androgen receptor blocker [54]. Since Akt initiates one of the primary pro-survival and anti-apoptotic pathways, the decrease in Akt signaling in males could compromise the normal mechanisms of tissue repair (Figure 4).

Therefore, it is not surprising that males that appeared to be under a higher risk of cell damage on the background of insufficient repair have activated pro-inflammatory signaling responsible for removing the dying cells (Figure 4). However, once activated, this inflammatory response can quickly become maladaptive and additionally promote the damage. Indeed, our research group has discovered that in males PAH associates with severe inflammatory and fibrotic changes in small pulmonary arteries and in RV that are not evident in females [15,20], Figure 4. It was also reported that testosterone promotes neutrophil infiltration in the myocardial infarct border zone. The increase in acute myocardial inflammation prevented myocardial healing after infarction and was proposed to be the reason for the increased cardiac rupture and death in testosterone-treated animals [56]. Males are also known to have a poor survival prognosis in acute inflammatory diseases. It was confirmed that the male gender is associated with more severe inflammation and was proposed to be an independent prognostic factor for infection-induced mortality [19]. Our recent research highlighted the importance of activation of pro-inflammatory pathways in the pathogenesis of PAH in males [15]. The increased tissue damage and inflammation are known triggers of pro-fibrotic changes required for maintaining vascular and cardiac integrity. Therefore, there is no surprise that testosterone also promoted maladaptive RV fibrosis [20,57], which in turn is linked to the poor survival prognosis in males.

Although the field is full of controversial reports regarding the role of sex hormones in pulmonary hypertension, the majority of previous research emphasizes the importance of estrogens and androgens in PAH pathogenesis. Nevertheless, their contribution to the disease is precisely different. While antioxidant protection seen in females could be responsible for apoptosis resistance and proliferative changes in pulmonary arteries (Figure 4), the preserved RV function ensures better survival prognosis. In contrast, in males the increased level of oxidative stress and reduced pro-survival pathways manifest as less severe vascular remodeling but more pronounced inflammation and fibrosis, both in pulmonary vasculature and in RV, which could reduce the survival rate (Figure 4). Based on these results, our team proposed two distinct PAH phenotypes—female “proliferative phenotype” and male “pro-inflammatory/pro-fibrotic phenotype” [20] that would require gender-specific therapeutic approaches for the appropriate and effective disease attenuation.

## 4. Sources of ROS in PAH and the Role of Gender

### 4.1. Mitochondrial ROS

Mitochondrial respiration is the primary source of ROS in the live cells. Although in a healthy state these ROS are precisely controlled, in disease, they may start playing a pivotal role due to either severely upregulated ROS production, or impaired antioxidant protection, or both. Thus, electron leak from the electron transport chain that produces superoxide is especially probable in conditions when NADH is supplied at a higher rate than the downstream reduction of oxygen can take place [58]. This situation was described to happen during apoptosis upon cytochrome c release or under conditions of hypoxia, both conditions directly related to PAH. Although there are a few sites of ROS emission in the mitochondria, the respiratory chain complexes I and III are considered to be the major sources of ROS [59]. However, while complex I generates superoxide on the matrix side, complex III could produce superoxide on both sides of the mitochondrial inner membrane, significantly shifting the mitochondria redox homeostasis, and through diffusion to the cytoplasm altering the activity of redox-sensitive proteins [41].

There is a strong sexual dimorphism in mitochondrial function. It is attributed to the exclusively maternal transmission of mitochondria that gets a better functional adaptation in females compared to males [55,60]. The basal and maximal respiration, the activity of the ETC, and ATP production were reported to be higher in females [61]. Mitochondria from female rats have higher mitochondrial DNA (mtDNA) and total protein content [62]. Although the overall mitochondrial content in female cardiomyocytes was found to be decreased, the increased functional capacity overcomes this difference [63]. Nevertheless, the highly efficient female mitochondria generate less ROS compared to males [64]. Besides, the higher content of antioxidant enzymes protects females from mitochondrial ROS more efficiently than males. Thus, the estrogen that mediated increase in SOD2 and glutathione peroxidase expression yields females greater protection during oxidative stress conditions [65,66]. In addition, estrogens control the expression of mitochondrial proteins by regulating the transcription of mitochondrial and nuclear genes. This, in turn, allows maintaining mitochondrial integrity and protect the cells from apoptosis induced by mitochondrial dysfunction [67]. Moreover, mitochondria also contain the enzymes responsible for steroidogenesis, in particular, for the synthesis of progesterone [68], which was proposed to additionally contribute to mitochondrial protection in females. Thus, administration of progesterone after traumatic brain injury rescued ovariectomy-induced mitochondrial oxidative stress and prevented decrease in mitochondrial respiration [69]. In another study, mitochondrial function in females was discovered to be independent of phases of the estrous cycle; thus, it was suggested that progesterone rather than E2 protects brain mitochondria in females [70].

When compared to females, males are generally viewed as more prone to mitochondrial impairment and oxidative stress, in particular, due to the less effective antioxidant protection [60]. The decreased mitochondrial function, impaired mitochondrial redox status, and increased mitochondrial calcium overload in males, was proposed to contribute to the increased cell damage and death associated with the male gender [60]. Yet, testosterone supplementation was shown to increase mitochondrial function and mitochondrial biogenesis, as well as to reduce mitochondrial oxidative stress [71,72]. In contrast, low levels of testosterone were associated with mitochondrial dysfunction, including impaired mitochondrial respiration and reduced expression and activities of metabolic enzymes [73]. This contradiction could be related to the combination of hormonal and genomic factors regulating mitochondrial function in both genders and requires additional research.

The fundamental role of mitochondria in the pathogenesis of pulmonary hypertension is currently widely accepted and was formulated as “metabolic theory of PAH” [74]. According to the current opinion, suppressed mitochondrial respiration and increased cytoplasmic glycolysis, or shift to the “cancer-like” glycolytic metabolism, stimulates proliferative capacity of the pulmonary vascular cells and mediates PAH progression [37]. For example, inhibition of pyruvate dehydrogenase (PDH), which converts pyruvate into acetyl-CoA in the mitochondria, is shutting mitochondrial electron transport chain down. This, in turn, reduces the generation of mitochondrial ROS [75]. The suppressed mitochondrial ROS (mROS) production was confirmed to significantly alter the downstream signaling pathways and promote PAH [76]. Thus, it induces inhibition of membrane potassium voltage-gated (Kv) channels [77], leading to an increase in the intracellular calcium with subsequent pulmonary vasoconstriction and proliferation of pulmonary vascular cells. Reduced mROS also activate hypoxia inducible factor HIF1α in the absence of hypoxia [78]. Stabilization of transcriptional factor HIF1α signifies cell pro-survival mechanisms that are well implicated in the pathogenesis of PAH [37]. Importantly, once started, this mechanism continues to amplify itself, promoting and prolonging the pathological events. Thus, the normoxic stabilization of HIF1α leads to the activation of PDH inhibiting enzyme, PDH kinase (PDK), producing a vicious cycle in mitochondrial redox disbalance and sustaining the PAH phenotype [75]. The similar shift of mitochondrial redox balance from oxidized to reduced was implemented in the pathogenesis of hypoxia-mediated PAH [79]. Therefore, one can conclude that mROS are important upholders of mitochondrial redox health. Although high ROS levels are known to be deleterious, the excessive depletion in mROS is also pathogenic and can promote PAH. Given that females have a significantly amplified mitochondrial antioxidant system, they could be expected to be more amenable to over-depletion in mROS, especially on the background of mitochondrial dysfunction, which could contribute to the predisposition of the female gender to PAH. Indeed, the increased basal level of HIF1α in female pulmonary vascular cells compared to males has been recently confirmed [80].

An alternative mechanism that could be involved in the higher female prevalence is related to lipid metabolism that was shown to be more active in females compared to males [81,82]. Indeed, in PAH an attenuation of PDH activity reduces the conversion of pyruvate to acetyl-Coenzyme A (CoA). With less acetyl-CoA available, the synthesis of malonyl-CoA, an inhibitor of fatty acid oxidation, is reduced and the metabolism switches to the fatty acid oxidation (FAO) [83]. This maladaptive increase in FAO was confirmed to reduce RV function in pulmonary artery banding (PAB) model, while inhibitors of FAO enhanced RV function [84]. The increased FAO is accompanied by elevated expression of carnitine palmitoyltransferase (CPT1), an enzyme considered to be the rate-limiting step in the oxidation of long-chain fatty acids and responsible for fatty acid flux in the mitochondria. Given that in females the expression of CPT1 [81] and its activity [85,86] was found to be elevated in an estrogen-dependent manner, females could be potentially predisposed to the changes in metabolism associated with PAH.

### 4.2. NADPH Oxidases

NADPH oxidases (Nox) are a group of multicomponent enzymes that transport electrons across the plasma membrane to generate O_2_^−^ and other ROS. Nox enzymes consist of five Nox enzymes (Nox1–5) and two Duoxes (Duox1 and 2), most of which are expressed in vascular cells [87]. This family of Nox enzymes generates ROS through an active enzymatic process and is capable of synthesizing high levels of ROS in a spatial and temporal manner [88], supporting the importance of the biological function of ROS. Nevertheless, the pathological role of ROS produced by Nox enzymes in PAH is also well established. Thus, the expression of Nox1 was found to be increased in MCT rat model [89], while antioxidant therapy mediated decrease in Nox1 expression attenuated RV hypertrophy. Elevated Nox1 expression was shown to stimulate pulmonary artery smooth muscle cell proliferation and migration in rat PAH model [90,91]. A recent study has also observed an upregulation of Nox1 expression and protein levels in resistant pulmonary vessels from PAH patients [92]. The increased ROS production upregulated the expression of Gremlin1, which functions as an antagonist of bone morphogenetic proteins BMP2 and BMP4, while silencing Nox1 gene attenuated Gremlin1-mediated signaling and hypoxia-mediated endothelial cell proliferation, suggesting the novel mechanisms of Nox1-dependent endothelial cells (EC) proliferation.

The contribution of Nox2 in PAH is supported by even a larger body of literature [87,93] and was shown to be involved in pulmonary artery vasoconstriction, smooth muscle remodeling, and RV hypertrophy [94,95]. Interestingly, hypoxia-induced activation and membrane translocation of p47phox subunit of Nox2 in pulmonary but not mesenteric arteries [96] suggests the unique function of Nox2 in hypoxic pulmonary vessels compared to the systemic circulation. Phosphorylation of gp91phox and p47phox, critical for Nox2 membrane translocation and assembly, is regulated by protein kinase C (PKC) [97], an important mediator of vascular cell proliferation. Since activation of NOX triggers the stimuli that activate PKC itself, this regulation could represent an example of the self-amplifying ROS/proliferation pathway promoting uncontrolled vascular growth in PAH.

There is also strong evidence of the direct involvement of Nox 4 in PAH [87,93]. It is expressed in all layers of pulmonary arteries and considered to be the major Nox isoform expressed in pulmonary artery smooth muscle cells (PASMC) [98]. The study that evaluated the expression of different Nox subunits in mouse lung tissue under hypoxic condition proposed that hypoxia exclusively induced an upregulation of Nox4 mRNA [99]. In addition to the numerous reports regarding the role of Nox4 in animal models [88], there is also evidence of significant elevation of Nox4 in lungs of patients with idiopathic pulmonary arterial hypertension (IPAH) [99,100].

Despite the significant progress in understanding the contribution of Noxs in PAH pathogenesis, there are also many contradictions. Thus, loss or overexpression of Noxs does not always alter PAH [101]. Moreover, knockout of Nox in mice was shown to promote PAH instead of mediating protection [102], and the phenotype was rescued back by Nox re-expression. It was noticed that some of the studies reporting the opposite outcomes were performed on mice of different genders [87]. Indeed, the contribution of gender in Nox activity was previously reported. For example, treatment of vascular EC with 17-estradiol inhibits Nox activity and ROS production [103]. The same effect was achieved in male spontaneously hypertensive rats treated with estrogen receptor agonist [104]. Miller et al. investigated the NADPH oxidase activity in the cerebral arteries of rats and found that the activity of Nox1 and Nox4 was 2–3 folds lower in females compared to males [105]. Ovariectomy (OVX) increased superoxide production, while treatment with exogenous estradiol restored it back. The particular influence on the Nox activity also depends on the type of sex hormone. Thus, in contrast to estradiol, progesterone can increase NOX activity and even antagonize the protective effect of E2 [106]. This suggests even more complex contributions of gender in NOX regulation since the different hormonal status could drive the opposite outcome. In males, testosterone stimulates NADPH oxidase activity and expression, increases NOX-derived ROS production in vascular cells, and induces smooth muscle cell (SMC) and leukocyte migration [44,107]. Thus, NOXs could play an important role in the manifestation of the male-specific phenotype described earlier, including an increased level of oxidative stress and activated pro-inflammatory response. Indeed, the study that directly compared superoxide production in microvessels of male and female hypertensive rats highlighted NOX-dependent elevation of oxidative stress in male rats [108].

### 4.3. Free Heme, Heme-Containing Proteins, and Free Iron

Heme is an ubiquitous co-factor of many enzymes; this tetrapyrrole is structurally classified into several types such as heme a, b, c, o, siro, d1, P460, and also ‘unusual heme’ [109]. Among these heme types, heme b is much more widely prevalent in animals. Heme-containing proteins, such as hemoglobin and myoglobin, perform pivotal roles in oxygen transport and storage. A host of heme-enzymes such as peroxidases, peroxygenases, oxidases, mono- and di-oxygenases have been researched intensely, and their far-reaching implications in several fundamental cellular processes such as respiration, electron transfer, detoxification, drug metabolism, redox homeostasis, secondary metabolite generation, and in cellular signaling have been well established. Heme-containing proteins are also involved in the generation of ROS as well as the detoxification of ROS [110]. Redox reactions are responsible for electron transfer and energy metabolism. Due to the central role of heme-containing proteins in redox reactions, their expression and concentrations are regulated stringently in an attempt to prevent the disruption of redox homeostatic balance. The ligands of heme iron in enzymes such as nitric oxide [111,112], superoxide [113], hydrogen peroxide, and hydrogen sulfide (H_2_S) [114] have been well explored in their ability to manifest cellular dysfunction in pathologies such as cancer, cardiovascular diseases, microbial persistence, etc. Thus, the regulation of heme-containing enzymes in pathobiological mechanisms of disease development and progression is extremely important.

Heme is considered to be a signaling molecule [115]. Upon binding to an antibody, heme was shown to acquire peroxidase activity [116]. The active sites of heme-containing proteins possess heme coordinated to iron in the Fe^2+^ or Fe^3+^ state. The binding of molecular oxygen or hydrogen peroxide may oxidize iron to form high valent iron (oxidation states of IV^+^ or V^+^) during the peroxidase as well as oxidase cycles [117]. Both one- and two-electron oxidations of the bound substrate(s) lead to oxidation of these substrates by the heme-containing enzymes. The cellular concentrations of the heme and iron (especially in Fe^2+^ state) are tightly regulated [118]. Iron is seldom present in the reduced (Fe^2+^) form due to its ability to degrade hydrogen peroxide via the Fenton reaction [119] to form the powerful hydroxyl radical, one of the most potent oxidants known to drive DNA and macromolecular damage [120]. Also, iron exists in a sequestered form, as in ferritin, or otherwise is bound to heme porphyrin or non-heme proteins or iron–sulfur cluster proteins. Therefore, in pathophysiological conditions, it is understood that oxidative stress could be exacerbated by the release of free iron and free heme from their sources.

It is well accepted that chronic hemolytic diseases are associated with an increased risk of pulmonary hypertension [121]. Recent findings from our group have revealed that hemolysis and subsequent free heme signaling could be a cause of PAH [122]. We demonstrated that increased free hemoglobin in PAH patients was correlated with the severity of the disease. We also showed that free heme could contribute to increased lung vascular permeability, immune cell infiltration, and perivascular edema, leading to the activation of the p38/HSP27 pathway that initiates PAH in rats. The overall roles played by heme-containing proteins and free heme and iron in redox homeostasis, inflammation, and metabolism can have important repercussions in the programming of events leading to PAH. Heme was shown to induce SMC proliferation and migration via NADPH oxidase. Heme also seemed to activate proliferative nodes like the MAPK and nuclear factor (NF-κB) pathways. In doing so, it also activated HO-1 expression [123]. Another important finding was that heme could potentially cause endothelial cell dysfunction via increased iron-mediated ROS [124]. Recent research also showed that free heme in cells could trigger endoplasmic reticulum (ER) stress [125] that was also found in patients with PAH [126]. Treating rodents with the chemical chaperone phenyl-butyric acid alleviated ER stress and reduced symptoms of PAH induced by hypoxia [127], indicating that free heme could initiate PAH through ER stress. Free heme could also bind to soluble guanylate cyclase (sGC) and interfere with the signaling of NO, and therefore could be implicated in PAH [128].

Iron is an important micronutrient that is required for growth as well as health. A lack of iron in the diet leads to the development of nutritional anemias. In humans, the normal levels of iron in both men (13.5–17.5 g/dL) and women (12.5–15.5 g/dL) are different; this could be owing to differences in metabolic rates between the two sexes. Also, the serum concentration of ferritin, a storage form of iron, is about 69 ng/mL in men but is usually about 35 ng/mL in normal women [129]. These differences arise only after puberty because iron levels are not significantly different in the sexes prior to puberty [130]. Also, in the late teen years, serum ferritin levels increased markedly in Blacks and Hispanics than their Caucasian counterparts at comparable age and sex [131]. Hemoglobin concentrations in women have been found to match their aged counterparts only close to ten years after menopause [132,133]. Women are more susceptible to iron loss and are at a greater risk for impaired iron balance [134]. Iron deficiency is considered to be the single most important cause of idiopathic pulmonary arterial hypertension (IPAH) [135]. Ferric carboxymaltose (≤1000 mg) supplementation to 20 IPAH patients enhanced exercise capacity and quality of life in a pilot study [136]. Diet also could play a key role, because the typical Western diet contains several substances which could interfere with iron absorption [137]. A recent study showed that intravenous iron supplementation in IPAH patients improved their exercise capacity and quality of life [138]. Having identified iron deficiency in pulmonary arterial hypertension, a study evaluating the therapeutic potential of iron supplementation in PAH patients is currently underway [139]. Importantly, circulating iron levels can regulate the expression and activity of HIF1α, a transcription factor which controls hypoxia responses of pulmonary vasculature [140]. The important cytokine in IPAH was found to be interleukin-6 (IL-6), which was known, in turn, to activate the release of hepcidin from the liver [141,142], which reduces the release of iron from storage sites in the cell. Interestingly, the most important biomarker in predicting patient survival in IPAH was red blood cells (RBC) distribution width (RDW) [143], which can correlate with increased hemolysis in patients. Therefore, we can conclude that both low and high iron/heme levels can contribute to the disease. Futhermore, there is a gender difference in iron/heme metabolism that can induce a known gender dimorphism in PAH pathobiology.

## 5. Nitric Oxide Synthase

The family of nitric oxide synthases (NOS) produce NO, an important signaling messenger that controls many cellular functions. Thus, NO produced by endothelial NOS (eNOS) mediates vasodilation and controls vascular homeostasis by possessing anti-inflammatory, anti-thrombotic, and anti-proliferative properties [144]. Nevertheless, the normal function of eNOS can be impaired by a deficiency of eNOS co-factors, such as tetrahydrobiopterin (BH4), shortage of eNOS substrate L-arginine, disruption of the dimeric eNOS complex, impaired expression or function of eNOS activity regulators, including calcium/calmodulin, caveolin, and HSP90, and the increased production of eNOS endogenous inhibitor, asymmetric dimethylarginine (ADMA), all of which are implicated in PAH. This altered eNOS function unusually referred to as “eNOS uncoupling,” produces superoxide instead of NO, thus contributing to vasoconstriction and oxidative damage of vascular cells [145].

More importantly, the simultaneous presence of uncoupled eNOS producing superoxide O_2_^−^ and normally working eNOS results in the formation of both O_2_^−^ and NO that react with each other at near the diffusion limit to form peroxynitrite, a highly reactive oxidative and nitrative agent. By inducing oxidative and nitrative posttranslational modifications of many critical cellular enzymes, peroxynitrite severely alters the cellular function. Thus, the absence of caveolin-1 (Cav1)-mediated negative regulation of eNOS in Cav1^−/−^ mice results in over-activation of eNOS and increased protein nitration in lungs and development of severe pulmonary hypertension [146]. At the same time, treatment of Cav1^−/−^ mice with NOS inhibitor, L-NAME or peroxynitrite scavenger manganese (III) tetrakis (1-methyl-4-pyridyl) porphyrin pentachloride (MnTMPyP) reversed the PAH phenotype, confirming the critical role of protein nitration in PAH development [147]. It was also confirmed that one of the potential targets of peroxynitrite is PKG, which has been found to be nitrated and inactivated in pulmonary vasculature of Cav1^−/−^ mice [147] and lung tissues from IPAH patients [146].

There are a number of self-amplifying loops promoting the ongoing nitrative stress in pulmonary hypertension (Figure 2). Thus, it was shown that peroxynitrite is capable to further uncouple eNOS [22]. Our research team confirmed that this happens due to the nitration of eNOS and could be effectively attenuated by suppressing the endothelin 1 signaling [21]. Nitration and the subsequent inactivation of the key mitochondrial antioxidant enzyme, SOD2, also contribute to elevated O_2_^−^/ONOO^−^ [21,23]. Over-activation of eNOS could also occur due to eNOS stimulation by protein kinase B (PKB) also known as Akt. Akt is a serine/threonine kinase, involved in cell growth, migration, metabolism, and proliferation [148]. By phosphorylating Ser1177 of eNOS [149], Akt stimulates eNOS activation, inducing an excessive NO production [150]. On a background of increased production of superoxide, elevated formation of NO contributes to protein nitration. Interestingly, we have recently discovered that Akt itself is amenable to tyrosine nitration [24]. Moreover, aside from many reports confirming that nitration inhibits the activity of enzymes, we found that the nitration of tyrosine Y350 activates Akt by mimicking Akt phosphorylation [24]. Since nitration, compared to phosphorylation, is a stable and irreversible posttranslational modification, it is expected to produce a sustained activation of Akt and amplification of nitrosative stress, as well as an activation of Akt-mediated proliferative pathways (Figure 2). Indeed, Akt and Akt-mediated downstream signaling have been confirmed to play a causative role in PAH, since the attenuation of Akt/mTOR axis was found to be sufficient to prevent hypoxia-induced PAH and pulmonary artery remodeling [151,152,153].

The activity of eNOS is known to be stimulated by estrogen through numerous genomic and non-genomic signaling mechanisms and results in elevated eNOS expression and activity [154,155,156] (Figure 2). The genomic regulation occurs through the activation of estrogen-response elements located within the promoter region of eNOS, one of the estrogen target genes. The non-genomic regulation is manifested through the activation of tyrosine kinase-MAPK and Akt signaling, the stimulation of HSP90 binding to eNOS, which is critical for eNOS dimerization, and the increase in the local calcium availability, ultimately leading to eNOS phosphorylation. Therefore, females express higher eNOS levels and are generally known to have higher NO production. This increased bioavailability of NO is considered to be a primary cause of protection against cardiovascular diseases in females. For example, an increased eNOS expression and enzymatic activity found in EC isolated from the female twin was shown to be responsible for the elevated EC migration, angiogenesis, and wound healing compared to the male twin [157].

However, on the background of oxidative stress, this protection could turn to become a malfunction due to effective scavenging of NO by superoxide. Moreover, the increased NO bioavailability in females could contribute to the pathogenesis of PAH in females. As in Cav1^−/−^ mice, the co-occurrence of elevated production of NO and O_2_^−^ could result in severe protein nitration and potentiate PAH in females. Indeed, our research team has recently confirmed that oxidative stress induced by chronic inhibition of complex III in the mitochondrial respiratory chain is sufficient to initiate PAH in healthy female rats [43]. It is well established that complex III is the main source of ROS in mitochondria [41], and its inhibition additionally enhances superoxide production. The repeated injections of the selective complex III inhibitor, Antimycin A (AA), to female rats induced a PAH that was characterized by a significant accumulation of nitrated proteins in the pulmonary tissue and correlated with disease progression. Besides, we confirmed that in female but not male PAH patients the levels of nitrated protein in circulation is elevated and correlated with the markers of PAH progression [43]. We conclude that female PAH patients can suffer from increased nitrosative stress, which, in turn, contributes to PAH.

## 6. The Antioxidant System in PAH and the Role of Gender

### 6.1. Gender Differences in SOD and Catalase

During the first step of antioxidant protection, superoxide dismutase (SOD), rapidly converts superoxide to H_2_O_2._ There are three different isoforms of SOD: cytosolic SOD1 (Cu–Zn SOD), mitochondrial SOD2 (Mn-SOD), and extracellular SOD3 (Fe-SOD); each of these plays an important role in the antioxidant protection of a specific cellular site. Decreased expression of SODs at the mRNA and protein levels was found to be involved in the pathogenesis of PAH [158,159,160]. Thus, SOD1 knockout mice have elevated right ventricular systolic pressure (RVSP) and pulmonary arterial remodeling [161], suggesting the importance of cytosolic O_2_^−^ in PAH pathogenesis. The contribution of superoxide generated in mitochondria was established even more thoroughly. The expression of SOD2 was reported to be decreased in the PASMC of PAH patients and Fawn-hooded rats (FHR) with PAH [160]. The intact PASMC treated with SOD siRNA was shown to obtain a hyperproliferative PAH phenotype and mitochondrial dysfunction while SOD2 overexpression or SOD-mimetic metalloporphyrin Mn(III)tetrakis (4-benzoic acid) porphyrin (MnTBAP) reverses this hyperproliferative phenotype both in vitro and in vivo [160,162]. The potential mechanism of SOD2 deficiency in PAH includes SOD2 polymorphism that alters SOD2 expression and significantly increases susceptibility to PAH [163] and epigenetic dysregulation [160]. Accumulated due to SOD2 insufficiency, superoxide scavenge NO and induce vasoconstriction that impairs systemic oxygenation. Therefore, it is not surprising that the intratracheal delivery of recombinant SOD showed an improvement in tissue oxygenation and a reduced oxidative stress in lambs with persistent pulmonary hypertension of the newborn [164].

The increased ROS and RNS are capable of significantly potentiating cell proliferation. Therefore, SOD2 was reported to be a potent tumor suppressor and inhibitor of cell proliferation in cancer [165,166] and pulmonary vascular cells [160,162]. In contrast, SOD2 insufficiency disrupts oxygen sensing in mitochondria and creates a pseudo-hypoxic environment responsible for the normoxic activation of HIF-1α [167]. Stabilization of HIF-1α, in turn, is considered to be one of the central events in the transformation of pulmonary vascular cells into highly proliferative cells, and is involved in calcium homeostasis, metabolic reprogramming, extracellular matrix reorganization, increased cell proliferation, and recruitment of progenitor cells [168,169,170]. Finally, the extracellular SOD3 was also confirmed to be an important mediator of redox homeostasis in control and PAH animals. Thus, deletion of SOD3 significantly exacerbated hypoxia-induced and MCT-induced PAH by elevating RV pressure and RV hypertrophy [158]. In contrast, EC-SOD overexpression attenuated chronic hypoxic PH, muscularization of small pulmonary vessels, and collagen deposition [171].

While SOD is an important regulator of redox homeostasis, the next enzyme in the antioxidant protection, catalase, which degrades H_2_O_2_ into oxygen and water, is not of any less importance. Indeed, the relatively inactive superoxide has a very limited ability to cross the cellular membrane, while H_2_O_2_, a highly active oxidant, can easily travel throughout the cell. Therefore, it is only when activities of SOD and catalase are tightly coupled in a functional tandem, that they effectively eliminate the major ROS in cells, and protect DNA, cell proteins, and lipids from the oxidative damage. However, uncoupling of the SOD/catalase cooperative activity could result in an uncontrolled accumulation of ROS. Thus, mice overexpressing SOD2 alone show exacerbated hypoxia-induced PAH, while mice with overexpressed catalase show disease attenuation [172]. We have also previously shown that treatment with SOD mimetic, Tempol, was not enough to attenuate protein oxidation, hemodynamic changes, and metabolic syndrome-associated renal injury [173]. This suggests that on the background of catalase inefficiency, SOD activity is not enough to provide the sufficient antioxidant protection. On the contrary, treatment with recombinant catalase enhanced SOD3 activity, impaired due to SOD3 oxidative modification, and reduced oxidative stress as well as improved oxygenation in lambs with persistent pulmonary hypertension of the newborn (PPHN) [174]. The increased generation of H_2_O_2_ was found to be responsible for the significant decrease in soluble guanylate cyclase expression and impaired vasodilation, while treatment of pulmonary arteries isolated from PPHN with catalase restored vasodilator responses to exogenous NO [175].

As discussed earlier, the expression of many antioxidant enzymes is under estrogen control, which is mediated by the interaction of estrogen receptors with estrogen-response elements in the promoter region of target genes. However, while expression and even activity of SOD2 and SOD3 were confirmed to be significantly increased by estrogen in many tissues, including vascular cells [66,176], there is an inconsistency in literature regarding the responsiveness of SOD1, or catalase, to estrogen stimulation. Thus, in healthy mammalian cells, not estrogen receptors, but rather other transcription factors were proposed to stimulate catalase activity [177]. Indeed, in vascular smooth muscle cells, SOD1 and catalase expressions were found insensitive to estrogen stimulation [176]. Catalase expression and circulation levels remained unaltered in females after salpingo-oophorectomy or after initiation of estrogen replacement therapy [178]. Nevertheless, all the major antioxidant proteins, including catalase and SOD1, were found to be decreased in the adipocytes of OVX rats with metabolic syndrome and were restored back with E2 supplementation [179]. In rat intraperitoneal resident macrophages, catalase activity was significantly reduced by OVX, while estrogen administration restored it [180]. Catalase activity in erythrocytes was also found to be dependent on the stage of the estrous cycle [181]. There is also a controversy in the capability of progesterone to affect antioxidant proteins. Thus, in vascular smooth muscle cells, progesterone time- and concentration-dependently reduced the expression and activity of SOD2 and SOD3 and antagonized the stimulatory effects of estrogens [106]. At the same time, progesterone was found to be a potent inducer of catalase activity in both normal and breast cancer cells [182].

Compared to females, males are generally known to show low levels of antioxidant enzymes [50], although it is unclear whether this gender difference is due to the absence of female sex hormone stimulation, or because of the pro-oxidant activity of testosterone. Thus, the activity of SOD2 and catalase in castrate mice was much greater than in intact males [183], suggesting the contribution of male sex hormones in the decreased antioxidant enzymes expression and activity. The same conclusion was made in a study that showed the administration of exogenous testosterone in mice with spinal cord injury significantly reduced SOD and glutathione peroxidase (GPx) activities [184]. Nevertheless, another study highlighted the protective role of testosterone by showing that testosterone therapy increases the activity of SOD and GPx in cardiomyocytes of castrated male mice [185].

Interestingly, aging was found to contribute to the gender difference in the antioxidant profile and even further amplified it. For example, with age the activity of catalase gets decreased in males and increased in females [186]. This discovery suggests the involvement of mechanisms other than sex hormones that are responsible for gender difference. These mechanisms could, at least in part, explain the controversy in studies investigating the roles of sex hormones. Moreover, given that the average age of patients diagnosed with PAH has significantly increased [187], this gender disparity in the aged population is especially important to study.

### 6.2. Selenium in Gender-Specific Redox Homeostasis

Selenium is a vital component of several redox proteins, like glutathione peroxidases (Gpx) and thioredoxins [188]. At the same time, the major storage protein for selenium, selenoprotein P (SeP), was shown to be implicated in the pathogenesis of PAH. Thus, it was discovered that the levels of SeP were significantly higher in PAH patients compared to healthy controls [189]. The same study has also found that in rodents, SeP enhances PASMC proliferation via resistance to apoptosis and mitochondrial dysfunction—factors known to be involved in the pathogenesis of PAH. These observations are in accordance with the other research showing that selenium supplementation in the cell culture media is required to maintain cancer cell proliferation and colony formation [190]. In contrast, selenium-deprived cells showed significantly higher levels of lipid peroxidation due to an insufficiency of selenium-dependent glutathione peroxidases (GPx). A significant drop in activity of GPx and SOD and subsequent impairment in redox-dependent reactions including NO metabolism was found in the lungs of IPAH patients [191]. Thus, selenium and selenium-containing enzymes are also involved in the narrow balance between protection, that could become redundant and promote cell over-growth, and under-protection that could induce cell damage.

**Figure 5:**
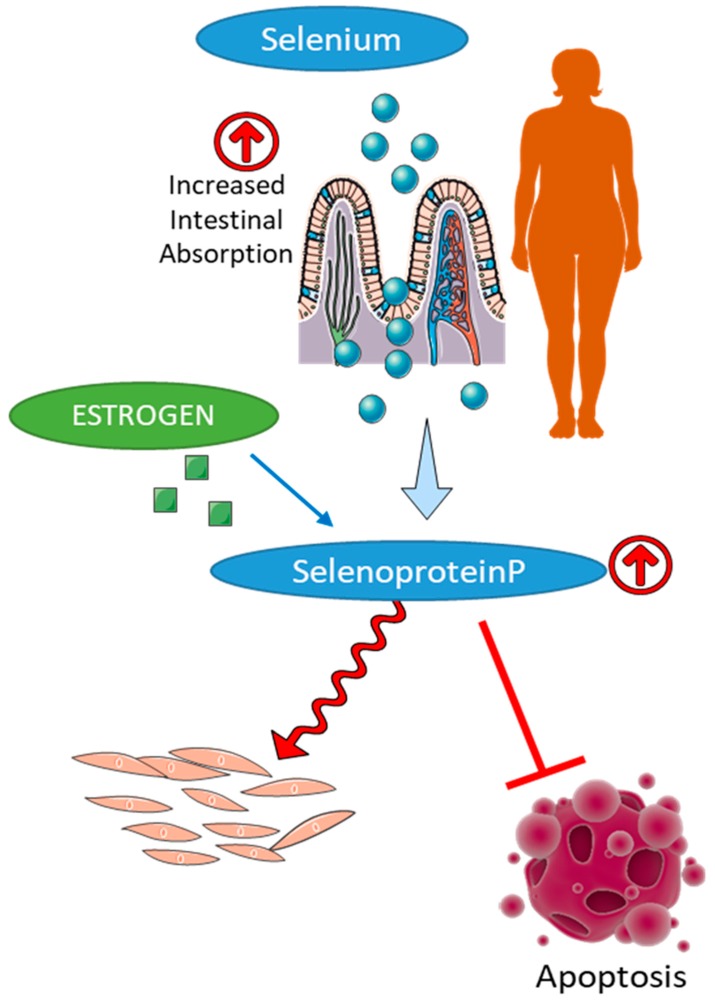
Regulation of selenium homeostasis in females. Female gender is associated with a higher level of selenium absorption, increased synthesis of selenoprotein P, and elevated activity of selenium dependent antioxidant enzymes, such as glutathione peroxidases and thioredoxins that contribute to the reduced cell damage and increased cell proliferation in females.

There is a remarkable difference in the plasma concentrations of selenium between males and females, owing to several reasons (Figure 5). In women, selenomethionine was shown to be absorbed to a greater extent (96%) than in men (76%) [192,193]. A similar trend was observed in the absorption of selenium in rodents [194]. Although the levels of total selenium could be the same in both genders, the distribution of selenium bound to albumin was found to be higher in females [195]. It was also shown that regardless of age, female rodents had higher levels of liver Selenoprotein P mRNA [196]. Females were reported to have an increased amount of circulating selenium and serum GPx activity [197], although male rodents had higher SeP levels in the kidney [196]. The decreased amount of selenium in males is proposed to be due to an intensive export of selenium to the testis at the expense of other tissues, while selenium deficiency in SelenoP^−/−^ mice was shown to make the mice infertile [194]. In contrast, in female rats, estrogen treatment increase selenium concentrations in the plasma, liver, and brain, and erythrocytes [198]. Estrogen treatment was shown to significantly increase GPx activity in the plasma and the liver [198]. At the same time, in OVX female rats, the expression GPx1 and GPx4 is decreased [199], suggesting that similar to SOD and catalase, GPx is estrogen dependent.

### 6.3. Gender Differences and Role of Heme Oxygenase in PAH

Heme oxygenase (HO) playing the primary role in the degradation of heme protects the cells from heme-mediated oxidative damage. There are two isoforms of heme oxygenase, inducible—HO-1 and constitutive—HO-2. HO-1 responds to a multitude of stress-inducing conditions such as hypoxia, hyperoxia, acidosis, shear forces, and reactive oxygen species (ROS) and, therefore, attracts more attention in the disease conditions [200]. Activation of HO-1 can remedy these stressors by producing anti-inflammatory, anti-apoptotic, and anti-proliferative effects on many different cell types [200]. These are particularly important in the vasculature remodeling seen in PAH. Aside from protecting cells against the pro-oxidant, heme, HO-1 is responsible for the production of the antioxidant, bilirubin. HO-1 also produces carbon monoxide (CO), which has been shown to stimulate guanylyl cyclase and increase intracellular levels of cGMP, resulting in vascular tone regulation [200]. The involvement of HO-1 was supported by the work of Belhaj et al. in a pre-clinical PAH model. Over a 6-month period, the piglets with a shunt from the left brachiocephalic artery to the pulmonary artery developed pulmonary vasculature changes and right ventricular hypertrophy, which correlated with decreased expression of HO-1 in the pulmonary tissue and decreased HO-1 activity in the right ventricle [201]. These findings support the idea that HO-1 is involved in protection against heme-induced changes in the vasculature, as we discussed previously.

HO-1 involvement in PAH was shown in papers dedicated to peroxisome proliferator-activated receptor (PPARγ) signaling. The PPARγ agonist, rosiglitazone, has been shown to dose-dependently stimulate HO-1 expression. Pulmonary artery smooth muscle cells (PASMC) that were given rosiglitazone showed a 12-fold increase in HO-1 levels [202]. Cells exposed to rosiglitazone also showed a dose-dependent reduction in proliferation. This supports the idea that HO-1 plays a major role in protecting against smooth muscle proliferation seen in PAH. This was further supported by the study performed by Zhang et al. that used rosiglitazone treatment in MCT rat model. The rosiglitazone treatment showed a significant decrease in right ventricular systolic pressure (RVSP) while also inhibiting right ventricular hypertrophy [203]. It also showed partial inhibition of pulmonary vascular remodeling. Thus, stimulation of HO-1 can protect the vasculature and resolve PAH.

HO-1 expression can also be affected by the time and duration of disease. In our study, which looked at how hemolysis and free heme can damage the endothelial cell barrier, the alterations of HO-1 at different time points of the disease were observed [122]. Our data indicated that HO-1 was markedly increased at the developed phase of disease in normoxic settings. During hypoxia treatment at 1 and 2 weeks of disease progression, the HO-1 levels did not increase, and in fact, the 2-week rats were found to have slightly decreased HO-1 levels. This was an unexpected result, as the oxidative stress caused by the hypoxia should be inducing HO-1 activity. We concluded that elevated free heme during hypoxia phase was a destabilizing factor for HIF-1a leading to the downregulation of HO-1.

The expression HO-1 was reported to be different in males and females. Thus, a study conducted by Macak-Safranko et al. looked at HO-1 activity in the liver of male and female mice subjected to oxidative stress in the hyperoxic chamber. They found that the amount of HO-1 mRNA was significantly increased in females while males did not change [204]. Another study by Pósa et al. also focused on differences in HO expression between males and females. They showed that Arginine vasopressin (AVP) increases HO-1 and HO-2 expression in the aorta and left ventricle of females compared to males [205]. These results support the idea that gender could alter the expression pattern of HO when exposed to stressful events and that HO-mediated gender dimorphism is involved in protecting cardiovascular systems to oxidative stress.

## 7. Conclusions

The concept of “redox homeostasis” includes a precise equilibrium between ROS and RNS production and the ability of the antioxidant system to efficiently eliminate their excess. Deviations such as ROS/RNS overage or deficiency promote a pathological state. We believe that a large body of accumulated evidence supports the note that redox homeostasis is gender-specific. In females, it is maintained by a highly active antioxidant defense; in males, however, the same balance is achieved through less dynamic protection. In a diseased state, this disparity predisposes two genders to different outcomes. Under moderate oxidative stress conditions, the better protected females experience less cell and tissue damage, although due to impaired mitochondrial respiration could suffer from ROS over-depletion that disrupts normal oxygen sensing and stimulates cell over-growth. The less protected males, however, experience more severe oxidative stress that promotes cell injury, inflammatory response, activation of fibrotic changes, and overall impairment of organ function. With excessive ROS production, which overruns antioxidant protection, females could suffer from a nitrative damage or nitration-mediated changes in cell homeostasis, occurring on a background of increased NO bioavailability. In contrast, males seem to be more protected from this type of stress. The particular mechanisms involved in the regulation of gender-specific stress response still remain to be clarified. Although on many occasions gender difference is attributed to the activity of sex hormones, the recent research started accumulating the knowledge about the contribution of genomic factors. Unfortunately, these studies dedicated to non-hormonal mechanisms of gender difference are limited due to historical interest in the effects of sex hormones, which overbalances the rest of the research. Nevertheless, some evidence clearly point toward the genomic-driven mechanisms that require extra attention [206]. The multiple levels of regulation add extra complexity and may explain the controversies in the field. We expect that future research will gain more clarity and significantly advance our understanding regarding the role of gender in the pathogenesis of PAH and other diseases. Nevertheless, even the currently accumulated knowledge is enough to conclude that the difference between the two genders in physiological and pathological responses requires gender-specific diagnostics and treatment approaches. This is especially important for diseases like PAH, with a well-established gender dimorphism.
